# *Terminalia chebula* Fruit Extract Ameliorates Peripheral Edema by Inhibiting NF-κB and MAPK Signaling Pathways

**DOI:** 10.3390/ijms26209965

**Published:** 2025-10-13

**Authors:** Sang-Hyup Lee, Sang-Yoon Kim, Yun-Gu Gwon, Su-Ha Lee, Ji-Soo Jeong, Je-Won Ko, Tae-Won Kim, Bong-Keun Choi

**Affiliations:** 1 NUON Co., Ltd., Seongnam 13201, Republic of Korea; lsh@nuonbio.co.kr (S.-H.L.); ksy5278@nuonbio.co.kr (S.-Y.K.); gyg@nuonbio.co.kr (Y.-G.G.); 2 BK21 FOUR Program, College of Veterinary Medicine, Chungnam National University, Daejeon 34134, Republic of Korea; suhai2729@gmail.com (S.-H.L.); jisooj9543@gmail.com (J.-S.J.); rheoda@cnu.ac.kr (J.-W.K.)

**Keywords:** peripheral edema, *Terminalia chebula*, vascular permeability, endothelial barrier, inflammatory response

## Abstract

Peripheral edema is a pathological condition caused by abnormal fluid accumulation in the interstitial space due to elevated vascular permeability and inflammation. This study evaluated the therapeutic efficacy of *Terminalia chebula* fruit extract (TCE) in inflammation-induced peripheral edema and clarified its molecular mechanisms. Using hydrogen peroxide (H_2_O_2_)-stimulated human umbilical vein endothelial cells (HUVECs), TCE was tested for effects on cell viability, inflammatory gene expression, intracellular reactive oxygen species, endothelial barrier integrity, and vascular endothelial growth factor (VEGF)-induced migration. Its influence on nuclear factor kappa-light-chain-enhancer of activated B cells (NF-κB) and mitogen-activated protein kinase (MAPK) signaling was examined. In vivo, TCE was assessed in acetic acid-induced peritoneal vascular permeability and carrageenan-induced paw edema models, followed by histological analysis and serum tumor necrosis factor-α (TNF-α) measurement. TCE restored cell viability (76.2% to 94.8%), reduced *TNF*, *IL6*, and *PTGS2* mRNA expression, and decreased reactive oxygen species by 27.2%. It enhanced barrier integrity, increased transendothelial electrical resistance, and inhibited VEGF-induced migration. TCE suppressed NF-κB and MAPK activation. In vivo, TCE reduced Evans blue extravasation by 41.6% and paw edema by 67.5%. Histology showed reduced dermal thickening and inflammatory infiltration, and serum TNF-α levels were lowered. TCE attenuates peripheral edema by preserving endothelial barrier function and suppressing inflammatory signaling, supporting its potential as a therapeutic agent for inflammation-associated vascular dysfunction and edema.

## 1. Introduction

Peripheral edema, which typically presents as swelling of the lower extremities, results from the abnormal accumulation of fluid in the interstitial space. It is frequently associated with impaired vascular integrity and increased capillary permeability, as observed in various clinical conditions including venous insufficiency and chronic inflammation [1,2]. Persistent edema can significantly affect quality of life by causing discomfort, reduced mobility, and skin complications such as ulceration or infection [3]. Despite its prevalence, treatment strategies primarily focus on symptomatic relief. Therefore, identifying agents that can regulate vascular function and modulate inflammatory pathways represents a critical need in edema-related research.

Inflammation acts as a primary mechanism underlying peripheral edema by engaging multiple interrelated signaling pathways. Pro-inflammatory cytokines, including tumor necrosis factor-α (TNF-α), interleukin-1β (IL-1β), and interleukin-6 (IL-6), markedly enhance vascular permeability, both through disruption of endothelial tight junctions and induction of vascular endothelial growth factor (VEGF) expression [4,5].

At the cellular level, these inflammatory mediators activate intracellular signaling cascades, mainly through the nuclear factor-κB (NF-κB) and mitogen-activated protein kinase (MAPK) pathways, leading to increased vascular permeability. Meanwhile, reactive oxygen species (ROS) generated under oxidative stress conditions further compromise endothelial barrier integrity [6,7,8,9]. This multifactorial disruption of endothelial function promotes fluid extravasation into interstitial tissues, resulting in peripheral edema.

The fruit of *Terminalia chebula* is one of the most valued medicinal plants in traditional Ayurvedic and Persian medicine, with documented use in treating various respiratory and digestive disorders. Pharmacological studies have substantiated the antioxidant properties of *Terminalia chebula* fruits, demonstrating effective scavenging of ROS in both in vitro and in vivo models [10,11,12,13]. These findings support its traditional use and suggest potential utility in inflammatory conditions, particularly those in which the accumulation of ROS plays a contributory role.

Our previous research demonstrated that *Terminalia chebula* fruit extract (TCE) effectively attenuated inflammation in osteoarthritis models by downregulating key inflammatory signaling pathways, including NF-κB and MAPK [14]. However, general anti-inflammatory strategies often fail to fully resolve peripheral edema, because the direct cause is localized endothelial barrier dysfunction that leads to vascular leakage and excessive fluid extravasation [15,16]. Evaluating the ability of TCE to protect endothelial integrity therefore provides a novel and clinically relevant perspective beyond its previously reported anti-inflammatory activity. To the best of our knowledge, its effects on key pathological features of peripheral edema, namely, vascular inflammation and fluid imbalance, have not yet been elucidated. Therefore, the present study aimed to evaluate the anti-edematous effects of TCE and investigate its underlying molecular mechanisms.

## 2. Results

### 2.1. High-Performance Liquid Chromatography (HPLC) Analysis of TCE

**Figure 1:**
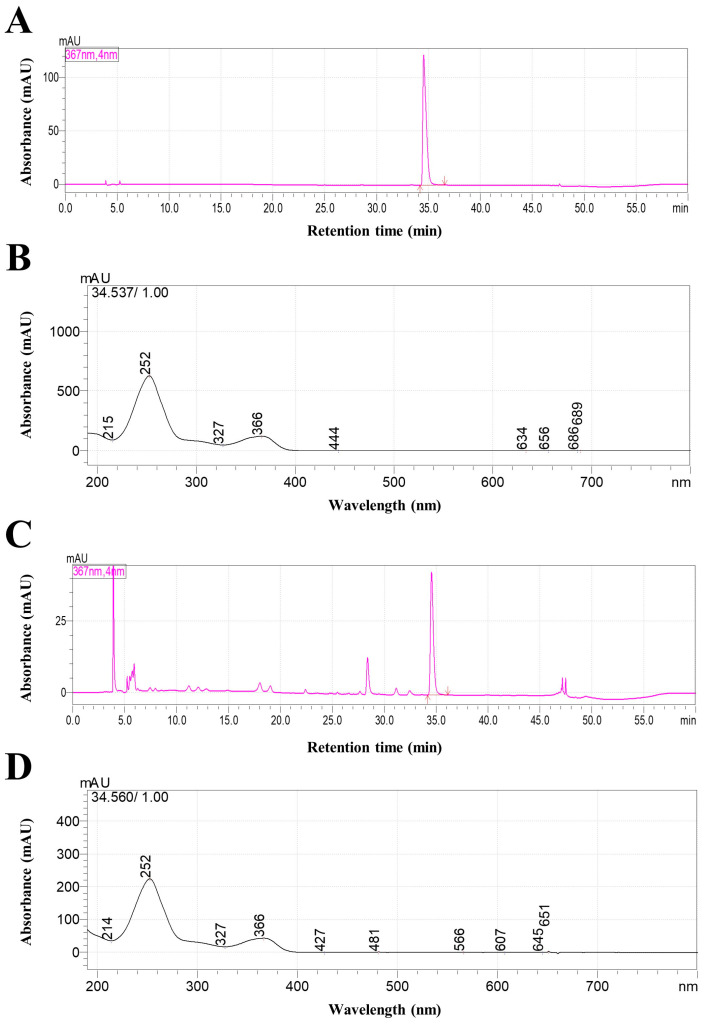
HPLC chromatograms and UV spectra of ellagic acid standard and TCE. (**A**,**B**) Chromatogram and UV spectrum of ellagic acid standard. (**C**) Chromatogram of TCE. (**D**) UV spectrum of the major peak in TCE.

A representative HPLC chromatogram of TCE is presented in Figure 1. Ellagic acid was clearly separated from other constituents in TCE, confirming its suitability as a reference marker compound, and was thus designated as a quality control marker for TCE. The retention time and UV spectrum of the corresponding peak were confirmed to be consistent with those of the ellagic acid standard.

### 2.2. TCE Restores Cell Viability and Suppresses Inflammation

**Figure 2:**
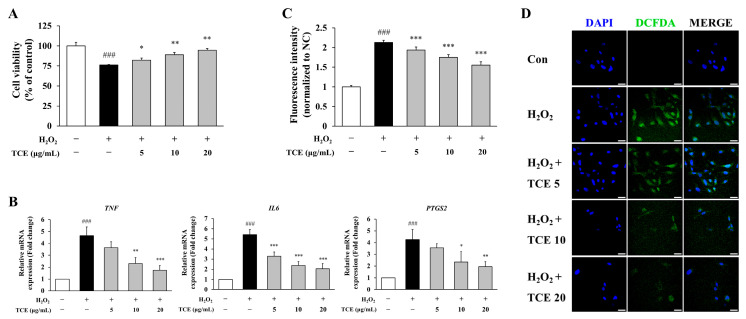
Protective effects of TCE against H_2_O_2_-induced endothelial damage and inflammatory responses in HUVECs. (**A**) Assessment of cell viability via MTT assay in HUVECs exposed to 100 μM H_2_O_2_, followed by treatment with 5, 10, or 20 μg/mL of TCE. (**B**) Quantitative analysis of mRNA expression of the pro-inflammatory mediators *TNF*, *IL6*, and *PTGS2* by qPCR. (**C**) Measurement of intracellular reactive oxygen species (ROS) through DCFDA assay in HUVECs. (**D**) Fluorescence microscopy images showing DCFDA-stained HUVECs (green) and nuclear counterstaining with DAPI (blue). Images were obtained at 63× magnification with a scale bar of 25 μm. Treatment groups are denoted as: TCE5, TCE 5 μg/mL; TCE10, TCE 10 μg/mL; TCE20, TCE 20 μg/mL. The data are presented as mean ± SD. Statistical significance is indicated as follows: * *p* < 0.05, ** *p* < 0.01, *** *p* < 0.001 compared to the H_2_O_2_-treated group; ### *p* < 0.001 compared to untreated control.

To assess the protective properties of *Terminalia chebula* fruit extract (TCE) against H_2_O_2_-induced endothelial damage, human umbilical vein endothelial cells (HUVECs) were exposed to 100 µM H_2_O_2_, followed by TCE treatment. H_2_O_2_ exposure significantly decreased cell viability to 76.2 ± 0.5% compared to the control group (*p* < 0.001), while subsequent treatment with 20 μg/mL TCE restored cell viability to 94.8 ± 2.0% (*p* < 0.01, Figure 2A).

To evaluate the anti-inflammatory effects of TCE, we quantified the mRNA expression levels of representative inflammatory markers, including *TNF*, *IL6*, and *PTGS2* (COX-2). At 20 μg/mL, TCE treatment effectively suppressed the expression of these targets under H_2_O_2_-induced condition by 62.6% (*p* < 0.001), 62.2% (*p* < 0.001) and 54.2% (*p* < 0.01), respectively (Figure 2B).

Furthermore, intracellular ROS accumulation, which serves as a crucial upstream activator of inflammatory signaling, was evaluated to further elucidate the anti-inflammatory mechanism of TCE. Quantitative analysis using DCFDA revealed that TCE significantly attenuated H_2_O_2_-induced ROS generation (*p* < 0.001). At a concentration of 20 μg/mL, TCE reduced ROS levels by 27.2% (*p* < 0.001) compared to the H_2_O_2_-treated group (Figure 2C). This finding was corroborated by diminished fluorescence intensity observed in DCFDA-stained cells (Figure 2D). Collectively, these findings indicate that TCE confers protective effects and exhibits anti-inflammatory properties under inflammatory conditions.

### 2.3. TCE Enhances Endothelial Barrier Function and Inhibits Inflammatory Activation in HUVECs

**Figure 3:**
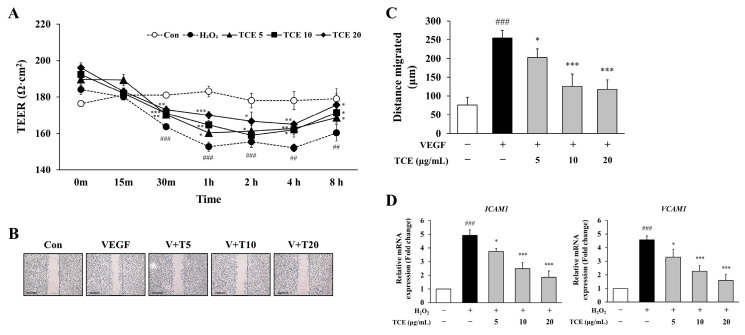
Effect of TCE on endothelial barrier integrity and VEGF-induced migration in HUVECs. (**A**) Evaluation of endothelial barrier integrity in HUVECs by TEER assay after treatment with 100 μM H_2_O_2_ and TCE at concentrations of 5 (TCE5), 10 (TCE10), and 20 μg/mL (TCE20), including untreated control (Con). The data are expressed as mean ± SD. Statistical significance is indicated as * *p* < 0.05, ** *p* < 0.01, *** *p* < 0.001 compared to the H_2_O_2_-treated group; ## *p* < 0.01, ### *p* < 0.001 compared to untreated control. (**B**) Assessment of VEGF-induced migration in HUVECs following TCE treatment. Microscopy images were obtained at 4× magnification, with a scale bar of 500 μm. Group abbreviations: Con, untreated control; VEGF, vascular endothelial growth factor; V + T5, VEGF with 5 μg/mL TCE; V + T10, VEGF with 10 μg/mL TCE; V + T20, VEGF with 20 μg/mL TCE. (**C**) Quantification of migration distance (μm) corresponding to (**B**). The data are expressed as mean ± SD. Statistical significance is indicated as * *p* < 0.05, *** *p* < 0.001 compared to the VEGF-treated group; ### *p* < 0.001 compared to untreated control. (**D**) Analysis of mRNA levels for adhesion molecules *ICAM1* and *VCAM1* in HUVECs following treatment with 100 μM H_2_O_2_ and TCE at 5, 10, or 20 μg/mL, as determined by qPCR. The data are expressed as mean ± SD. Statistical significance is indicated as * *p* < 0.05, *** *p* < 0.001 compared to the H_2_O_2_-treated group; ### *p* < 0.001 compared to untreated control.

To assess the impact of TCE on endothelial barrier integrity, the transendothelial electrical resistance (TEER) assay was conducted in HUVECs following H_2_O_2_ exposure. TCE treatment effectively restored TEER values in a concentration-dependent manner, indicating improved integrity of the endothelial monolayer. One hour after treatment, TCE at 20 μg/mL elevated TEER values to 170.0 ± 2.0, compared to 152.7 ± 2.5 observed in the H_2_O_2_ group. This improvement persisted at 8 h, with the TCE 20 μg/mL group exhibiting TEER values of 175.7 ± 6.0 (Figure 3A).

To investigate the regulatory effects of TCE on HUVECs, we assessed VEGF-induced cell migration as a representative model for pathological endothelial activation. Stimulation with VEGF significantly promoted migration of HUVECs, as measured by the mean migrated distance (254.3 ± 20.8 μm). Treatment of TCE led to a substantial reduction, decreasing VEGF-induced migration by 53.8% at a concentration of 20 μg/mL (*p* < 0.001), indicating inhibition of endothelial hyperactivation that frequently occurs during angiogenic and inflammatory conditions. Since VEGF not only promotes physiological angiogenesis but also contributes to pathological vascular remodeling and inflammation, inhibition of VEGF-induced migration by TCE supports its potential in maintaining vascular homeostasis (Figure 3B,C). Furthermore, TCE significantly downregulated the mRNA expression of adhesion molecules *ICAM1* and *VCAM1* (Figure 3D).

### 2.4. TCE Attenuates Activation of NF-κB and MAPK Signaling Pathways in HUVECs

**Figure 4:**
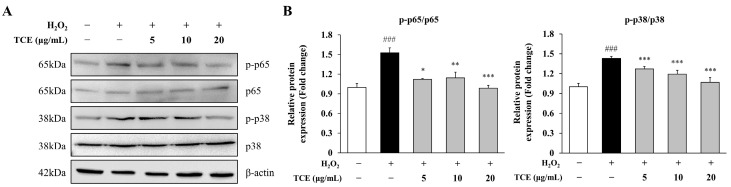
Mechanism of action of TCE on NF-κB and MAPK signaling pathways in HUVECs. (**A**) Western blot analysis demonstrating NF-κB p65 and MAPK p38 phosphorylation in HUVECs treated with 100 μM H_2_O_2_ and various concentrations of TCE (5, 10, or 20 μg/mL). (**B**) Quantification of phosphorylated NF-κB p65 and MAPK p38, each normalized to their respective total protein levels as shown in (**A**). The data are presented as mean ± SD. Statistical significance is indicated as * *p* < 0.05, ** *p* < 0.01, *** *p* < 0.001 compared to the H_2_O_2_-treated group; ### *p* < 0.001 compared to untreated control.

NF-κB and MAPK signaling cascades play critical roles in endothelial inflammation. To determine the involvement of these pathways in TCE-mediated anti-inflammatory effects, we analyzed the phosphorylation of p65 and p38 in HUVECs. H_2_O_2_ stimulation led to a significant rise in p65 phosphorylation (1.5-fold) compared to the control, indicating activation of the NF-κB pathway. TCE at 20 μg/mL effectively suppressed this elevation, restoring p65 phosphorylation to levels comparable to the control (1.0-fold). A similar effect was observed for p38, which increased to 1.4-fold after H_2_O_2_ exposure but was reduced to 1.1-fold with TCE treatment (Figure 4A,B). These results indicate that TCE suppresses inflammatory signaling by selectively inhibiting the activation of NF-κB and MAPK pathways.

### 2.5. TCE Reduces Vascular Permeability and Inflammation-Induced Paw Edema

**Figure 5:**
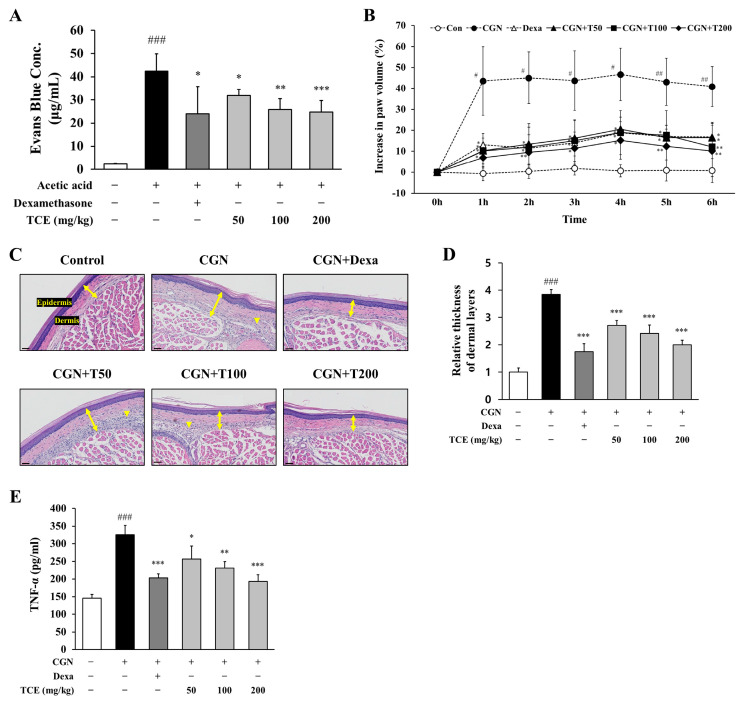
Evaluation of TCE effects on vascular permeability and inflammation in vivo. (**A**) Assessment of Evans blue dye extravasation in the peritoneal cavity following acetic acid-induced vascular permeability. TCE was administered at indicated doses. Dexamethasone (2 mg/kg, i.p injection) served as the positive control. The data are presented as mean ± SD. Statistical significance is indicated as * *p* < 0.05, ** *p* < 0.01, *** *p* < 0.001 compared to the acetic acid-induced group; ### *p* < 0.001 compared to untreated control. (**B**) Paw edema volume measured in the carrageenan-induced inflammation model after TCE treatment. Dexamethasone (2 mg/kg, i.p. injection) served as the positive control. The data are presented as mean ± SD. Statistical significance is indicated as * *p* < 0.05, ** *p* < 0.01 compared to the CGN-induced group; # *p* < 0.05, ## *p* < 0.01 compared to untreated control. (**C**) H&E-stained sections of paw tissue. Images were obtained at 30× magnification. Double-headed arrows indicate dermal thickness, and arrowheads denote inflammatory cell infiltration. Scale bars = 40 μm. (**D**) Quantification of dermal thickness. The data are presented as mean ± SD. Statistical significance is indicated as *** *p* < 0.001 compared to the CGN-induced group; ### *p* < 0.001 compared to untreated control. (**E**) Serum levels of TNF-α measured by ELISA in the carrageenan-induced inflammation model. The data are presented as mean ± SD. Statistical significance is indicated as * *p* < 0.05, ** *p* < 0.01, *** *p* < 0.001 compared to the CGN-induced group; ### *p* < 0.001 compared to untreated control. Abbreviations: CGN, carrageenan; Dexa, dexamethasone; T50, TCE 50 mg/kg; T100, TCE 100 mg/kg; T200, TCE 200 mg/kg.

To determine whether TCE exerts a protective effect against vascular dysfunction in vivo, we conducted an acetic acid-induced peritoneal vascular permeability assay. Vascular permeability was evaluated by measuring Evans blue dye extravasation into the peritoneal cavity. Injection of acetic acid led to a substantial increase in Evans blue leakage (42.5 ± 7.4 μg/mL), indicating enhanced vascular leakage during inflammation. Administration of TCE markedly lowered Evans blue extravasation to 24.8 ± 5.0 μg/mL corresponding to a 41.6% decrease compared to the acetic acid group (*p* < 0.001, Figure 5A).

The effect of TCE on peripheral edema was examined using a carrageenan-induced paw edema model. Carrageenan injection resulted in a pronounced elevation of paw volume, reaching a maximum at 4 h (46.7 ± 11.3%), demonstrating acute inflammatory edema. TCE administration at 200 mg/kg significantly reduced carrageenan-induced paw edema to 15.2 ± 9.0% (*p* < 0.05), a response similar to that of the positive control (dexamethasone, 18.8 ± 14.6%, *p* < 0.05), indicating that TCE effectively limits edema development following inflammatory stimulation (Figure 5B).

Inflammatory changes in carrageenan-induced paw edema tissue were subsequently analyzed. H&E staining demonstrated marked dermal thickening and increased infiltration of inflammatory cells. Treatment with TCE substantially diminished these histopathological changes, with the highest dose showing efficacy comparable to dexamethasone (Figure 5C,D).

To assess the systemic inflammatory response related to carrageenan-induced edema, serum levels of TNF-α were measured. Carrageenan treatment significantly elevated serum TNF-α levels (325.1 ± 27.3 pg/mL), while TCE at 200 mg/kg decreased these levels to 193.3 ± 19.0 pg/mL (*p* < 0.001), indicating that the anti-edematous activity of TCE is accompanied by reduction in pro-inflammatory cytokine on a systemic level (Figure 5E).

Together, these results provide in vivo evidence that TCE not only reduces vascular permeability but also attenuates peripheral edema by suppressing systemic inflammation, supporting its potential as a therapeutic agent for inflammation-associated vascular dysfunction and edema.

### 2.6. TCE Alleviates Tissue Inflammation by Modulating Cytokines and Inflammatory Signaling

**Figure 6:**
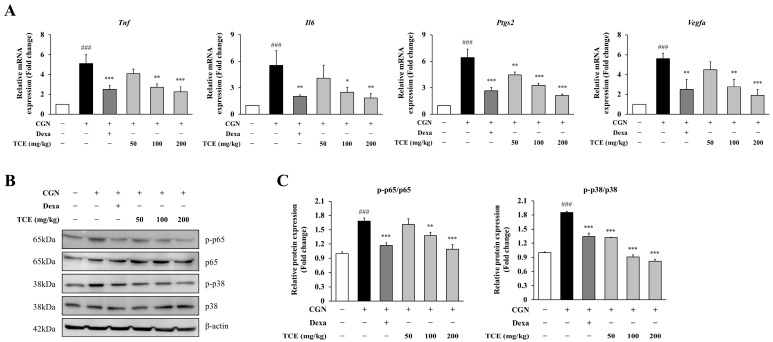
Molecular analysis of inflammation in the carrageenan-induced paw edema model. (**A**) Quantitative PCR analysis of *Tnf*, *Il6*, *Ptgs2* and *Vegfa* mRNA expression levels in paw tissue. (**B**) Western blot analysis of NF-κB p65 and MAPK p38 phosphorylation in paw tissue. (**C**) Quantification of phosphorylated NF-κB p65 and MAPK p38 normalized to total protein levels from panel (**B**). The data are presented as mean ± SD. Statistical significance is indicated as * *p* < 0.05, ** *p* < 0.01, *** *p* < 0.001 compared to the CGN-induced group; ### *p* < 0.001 compared to untreated control. Abbreviations: CGN, carrageenan; Dexa, dexamethasone.

To investigate the molecular effects of TCE, we measured mRNA expression levels of key inflammatory mediators in paw tissue. Carrageenan injection led to a marked upregulation of *Tnf*, *Il6*, *Ptgs2* (COX-2), and *Vegfa* (*p* < 0.001), reflecting transcriptional activation associated with acute inflammation. Notably, TCE administration at 200 mg/kg effectively suppressed the expression of these genes by 55.7% (*Tnf*), 66.9% (*Il6*), 66.8% (*Ptgs2*), and 65.9% (*Vegfa*) compared to the carrageenan group, demonstrating a suppressive effect similar to that observed with the dexamethasone-treated group (Figure 6A).

Consistent with these observations, we further examined the activation of NF-κB and MAPK signaling pathways. Phosphorylation of p65 and p38 was increased in the carrageenan-treated group, whereas TCE treatment markedly suppressed phosphorylation of p65 by 35.2% and p38 by 55.8% (at 200 mg/kg), without affecting total protein expression (Figure 6B,C). These results indicate that TCE alleviates tissue inflammation at both histological and molecular levels by inhibiting pro-inflammatory signaling pathways in vivo.

## 3. Discussion

Peripheral edema occurs in both acute and chronic inflammatory conditions and is often accompanied by pain, discomfort, and impaired mobility. It develops when inflammation compromises endothelial barrier integrity, resulting in increased vascular permeability and subsequent plasma extravasation into tissue [3,17,18]. Pro-inflammatory cytokines such as TNF-α and IL-6 promote disassembly of endothelial junctions by modulating tight junction components and upregulating adhesion molecule expression [19,20,21,22,23]. COX-2 further compromises barrier integrity through prostaglandin synthesis [24,25], whereas VEGF promotes cytoskeletal rearrangement and junctional disassembly, collectively contributing to vascular leakage and edema formation under inflammatory conditions [26,27,28].

The fruit of *Terminalia chebula* has long been used in traditional medicine for inflammatory and metabolic disorders. It exhibits potent antioxidant properties by suppressing reactive oxygen and nitrogen species [29]. We previously demonstrated its anti-osteoarthritic effects in IL-1β-stimulated human chondrocytes and in a rat model of monosodium iodoacetate–induced osteoarthritis, showing significant reductions in cartilage degradation and inflammatory markers. These findings support its traditional use and therapeutic potential for osteoarthritis [14]. Moreover, in line with its traditional use for fluid retention, which is commonly associated with vascular leakage and edema, a previous study reported that *Terminalia chebula* extract exhibits anti-edematous effects in a carrageenan-induced model [30]. However, that study primarily focused on overall symptomatic outcomes without addressing the underlying molecular mechanisms.

*Terminalia chebula* fruit contains diverse phytochemicals, including phenolic acids, tannins, lignans, and flavonoids [31]. Several of these compounds have been shown to exert antioxidant and anti-inflammatory effects by suppressing NF-κB phosphorylation, inhibiting MAPK activation, and modulating mediators such as TNF-α and VEGF (Table 1). These bioactive constituents are likely to act together to produce the anti-edematous effects of TCE.

These known anti-inflammatory mechanisms suggest that TCE may also be effective in reducing peripheral edema through similar pathways. While previous studies have mainly described the effects of *Terminalia chebula* on symptomatic outcomes, our work aimed to address this limitation by investigating its actions on inflammatory pathways and endothelial function. In this study, our findings collectively demonstrate that TCE confers protection against inflammation-induced endothelial dysfunction and peripheral edema. Using HUVEC-based cellular assays, we observed that TCE enhances endothelial barrier integrity and suppresses pro-inflammatory signaling. It also reduces leukocyte adhesion molecule expression and inhibits VEGF-induced endothelial migration, a process associated with pathological vascular remodeling [46]. These effects were consistently associated with inhibition of NF-κB and MAPK signaling pathways. Since NF-κB and MAPK signaling are well-recognized pathways involved in endothelial barrier disruption and vascular permeability [47,48], the inhibition observed in HUVECs provides a rationale for evaluating these effects in animal models.

For the in vivo experiments, TCE dosage range was determined based on the safety and efficacy results established in our previous report [14], applying interspecies dose conversion. TCE markedly suppressed vascular leakage in an acetic acid-induced peritoneal vascular permeability model, indicating its protective effect on endothelial barrier integrity. TCE also significantly reduced paw edema in a carrageenan-induced acute inflammation model, providing further evidence of its anti-inflammatory effect. Previous studies demonstrated that carrageenan-induced inflammation is not restricted to local effect but also induces systemic inflammation responses, including elevated pro-inflammatory cytokines such as TNF-α in serum levels [49,50,51]. Consistent with this, the reduction in serum TNF-α levels observed following TCE treatment supports its potential as a therapeutic agent for modulating systemic inflammation.

Taken together, our findings highlight that TCE protects against endothelial dysfunction and peripheral edema through suppression of NF-κB and MAPK signaling, restoration of barrier integrity, and reduction in systemic inflammation. These results provide both cellular and in vivo evidence for its therapeutic potential. Although TCE demonstrated beneficial effects in acute models of inflammation-induced edema, the present study is limited to short-term, chemically induced animal models that may not fully recapitulate the complexity of chronic edema. Peripheral edema in clinical settings is often associated with chronic inflammatory diseases such as chronic venous insufficiency and lymphedema, as well as metabolic disorders including obesity and diabetes [52,53,54,55]. Therefore, further studies are warranted to assess the long-term efficacy, safety, and pharmacological properties of TCE in chronic and clinically relevant models to better evaluate its therapeutic potential in long-term edema management.

## 4. Materials and Methods

### 4.1. Preparation of Terminalia chebula Fruit Extract and HPLC Analysis

*Terminalia chebula* fruits were extracted with water at 30–40 °C for 3–4 h under 1 bar pressure. The filtrate was concentrated under reduced pressure at 40–60 °C for 9–10 h, followed by spray-drying. HPLC was performed for marker compound analysis, with ellagic acid quantified as a reference marker. The content of ellagic acid in TCE was determined to be within 80–120% of the reference value of 13.8 mg/g.

### 4.2. Cell Culture

Primary human umbilical vein endothelial cells (HUVECs; PromoCell, Heidelberg, Germany) were cultured in endothelial basal medium (PromoCell, C-22210) supplemented with an endothelial cell growth supplement mix (PromoCell, C-39215). The cells were maintained at 37 °C in a humidified atmosphere containing 5% CO_2_. In vitro studies were performed using HUVECs at passages less than 5.

### 4.3. Cellular Reactive Oxygen Species (ROS) Assay

Intracellular ROS levels were quantified using the cell-permeant probe 2′,7′-dichlorofluorescin diacetate (DCFDA; Abcam, Cambridge, MA, USA). HUVECs were seeded at a density of 4 × 10^4^ cells/well in 100 μL in a 96-well plate and incubated overnight at 37 °C with 5% CO_2_. The cells were treated with TCE at concentrations of 5, 10, or 20 μg/mL for 4 h, followed by exposure to 100 μM hydrogen peroxide (H_2_O_2_; Samchun, Pyeongtaek, Republic of Korea) for 1 h. After treatments, the cells were washed with assay buffer, incubated with 20 μM DCFDA for 30 min at 37 °C, washed again, and fluorescence was measured using a VICTOR Nivo^TM^ multimode plate reader (PerkinElmer Inc., Waltham, MA, USA) with excitation/emission at 485 nm/535 nm.

Additionally, HUVECs were seeded onto 8-well culture slides at a density of 4 × 10^4^ cells/well in 300 μL and incubated overnight under identical conditions. Cells were then treated with TCE (5, 10, or 20 μg/mL) for 4 h, followed by exposure to 100 μM H_2_O_2_ for 1 h. As described above, the cells were washed with assay buffer, incubated with 20 μM DCFDA, fixed using 4% paraformaldehyde, and mounted with DAPI-containing mounting solution. The stained cells were visualized with a confocal microscope (Leica Microsystems, Wetzlar, Germany) at excitation wavelengths of 405 nm (DAPI) and 488 nm (DCFDA). Experiments were independently performed three times.

### 4.4. Transepithelial Electrical Resistance (TEER) Assay

HUVECs were seeded at a density of 3 × 10^5^ cells/well in 400 μL in a 12-well plate containing transwell inserts with 0.4 μm pore membranes. The cells were cultured in endothelial cell growth medium supplemented with 5% fetal bovine serum. The medium was changed every 2 days, and TEER measurements were performed on day 7, once a confluent HUVEC monolayer had formed. The monolayer was treated with TCE (5, 10, or 20 μg/mL) and incubated for 4 h, followed by exposure to 100 μM H_2_O_2_. Transmembrane resistance was then determined using a Millicell-ERS voltammeter (Millipore Corporation, Billerica, MA, USA). Experiments were independently performed three times.

### 4.5. VEGF-Induced Migration Assay

HUVECs were maintained in a 6-well plate for 3 days. A linear scratch (500 μm in width) was generated using a scratcher tip (SPL, 201924, Pocheon, Republic of Korea). Cells were subsequently rinsed three times with DPBS (Welgene, LB 001-02, Gyeongsan, Republic of Korea) to eliminate detached cells and cellular debris. The wells were categorized into the following groups: control, VEGF (10 ng/mL; Gibco, 100-20-10UG), and VEGF (10 ng/mL) combined with TCE at concentrations of 5, 10, or 20 μg/mL. After 24 h of incubation, VEGF-induced cell migration was assessed using an inverted microscope (Eclipse Ts2, Nikon, Tokyo, Japan), and the migration distance was quantified by measuring at 5 randomly chosen positions along the scratch line per well using NIS-Elements software (version 5.00; Nikon). Experiments were independently performed three times.

### 4.6. Western Blot Analysis

HUVECs and mouse paw tissues were lysed in RIPA buffer supplemented with protease and phosphatase inhibitors for Western blot analysis. The antibodies employed in this study include anti-phospho-p65 (Cell Signaling, 3033S, Danvers, MA, USA), anti-p65 (Cell Signaling, 8242S), anti-phospho-p38 (Cell Signaling, 4511S), anti-p38 (Cell Signaling, 8690S), and anti-β-actin (Cell Signaling, 8457S). Western blot analysis was performed three times for HUVECs, and tissue samples from five mice per group (*n* = 5) were analyzed.

### 4.7. Quantitative RT-PCR

Total RNA was isolated from HUVECs using the RNeasy mini kit (Qiagen, 74104, Hilden, Germany) and reverse-transcribed into cDNA using a reverse transcriptase kit (Cellscript, CDS-400, Seoul, Republic of Korea). Quantitative real-time PCR was carried out with QuantStudio 3 (Applied Biosystems, A28567, Waltham, MA, USA). The following primers for human genes were applied: GAPDH (Forward, 5′-TGG TGC TGA GTA TGT CGT GGA GT-3′; Reverse, 5′-AGT CTT CTG AGT GGC AGT GAT GG-3′), TNF (Forward, 5′-CCC AGG CAG TCA GAT CAT CTT C-3′; Reverse, 5′-AGC TGC CCC TCA GCT TGA-3′), IL6 (Forward, 5′-GGT ACA TCC TCG ACG GCA TCT-3′; Reverse, 5′-GTG CCT CTT TGC TGC TTT CAC-3′), PTGS2 (Forward, 5′-TGG GAA GCC TTC TCT AAC CTC TCC T-3′; Reverse, 5′-CTT TGA CTG TGG GAG GAT ACA TCT C-3′), ICAM1 (Forward, 5′-CGA TGA CCA TCT ACA GCT TTC CGG-3′; Reverse, 5′-GCT GCT ACC ACA GTG ATG ATG ACA A-3′), VCAM1 (Forward, 5′-GAT ACA ACC GTC TTG GTC AGC CC-3′; Reverse, 5′-CAG TTG AAG GAT GCG GGA GTA TAT G-3′). For mouse genes, the following primers were included: GAPDH (Forward, 5′-GAA GGT CGG TGT GAA CGG AT-3′; Reverse, 5′-AGT GAT GGC ATG GAC TGT GG-3′), TNF (Forward, 5′-TTG ACC TCA GCG CTG AGT TG-3′; Reverse, 5′-CCT GTA GCC CAC GTC GTA GC-3′), IL6 (Forward, 5′-GCC TTC TTG GGA CTG ATG CT-3′; Reverse, 5′-TGG AAA TTG GGG TAG GAA GGA C-3′), PTGS2 (Forward, 5′-TGA GTA CCG CAA ACG CTT CT-3′; Reverse, 5′-TGG GAG GCA CTT GCA TTG AT-3′), VEGFA (Forward, 5′-TGT GTG AAG GTG CAG TTT TG-3′; Reverse, 5′-ATT TCT GTG TTG GCG CAG T-3′). All quantitative RT-PCR were performed in triplicates. The final data were derived from three independent biological experiments for HUVECs, and from tissue samples of five mice per group (*n* = 5) for mouse genes.

### 4.8. Experimental Animals

6-week-old male C57BL/6 mice were purchased from Samtako (Osan, Republic of Korea) and maintained in a clean, well-ventilated animal facility with controlled environmental conditions: temperature (23 ± 3 °C), relative humidity (30−70%), a 12 h light/dark cycle, and 10−20 times air changes per hour. Mice were acclimated for one week before experiments and then randomly assigned to six groups (*n* = 5 per group): control, acetic acid or carrageenan only, dexamethasone (2 mg/kg), and TCE (50, 100, or 200 mg/kg). The TCE dose range was determined based on our previous study in a rat model of inflammation [14], where efficacy and safety were demonstrated at 25–100 mg/kg. Using body surface area-based interspecies conversion, equivalent doses for mice were calculated to be approximately 50, 100, and 200 mg/kg. A 4-day dosing regimen was used to evaluate acute inflammatory responses. The sample size was based on results from preliminary studies. Both group allocation and data collection were performed in a blinded manner. All animal procedures were conducted in accordance with the guidelines of the Institutional Animal Care and Use Committee of Chungnam National University (approval No. 202503A-CNU-045 and -046).

### 4.9. Acetic Acid-Induced Peritoneal Vascular Permeability Mouse Model

TCE was administered orally once daily for 4 days, while dexamethasone was administered via intraperitoneal injection under the same schedule. One hour after the last administration, all mice received a tail vein injection of 200 μL of 2% Evans blue dye in saline. After 30 min, 200 μL of 0.6% acetic acid in saline was injected intraperitoneally to induce vascular permeability. The peritoneal cavity was washed with 5 mL of saline, and the lavage fluid was collected and centrifuged at 3000 rpm for 5 min. The absorbance of the supernatant was measured at 610 nm using a microplate reader to determine the amount of extravasated Evans blue dye.

### 4.10. Carrageenan-Induced Paw Edema Mouse Experiment

TCE was administered orally once daily for 4 days, while dexamethasone was administered via intraperitoneal injection under the same schedule. One hour after the last dose, 50 μL of 1% carrageenan in saline was injected subcutaneously into the plantar surface of the right hind paw to induce acute inflammation. Paw thickness was measured every hour for 6 h using a plethysmometer.

### 4.11. Histological Analysis

Paw tissues were fixed in 10% formalin for 4 h immediately after harvesting, followed by fixation in 10% formic acid prepared in 10% formalin for 5 days. The formic acid-formalin solution was replaced daily. For complete decalcification, the tissues were treated with Decalcifying Solution-Lite (Sigma-Aldrich, St. Louis, MO, USA) at a tissue-to-solution ratio of 1:20 (*v*/*v*) for 4 h, then rinsed under running tap water for 4 h. Samples were then processed, embedded in paraffin, and sectioned at a thickness of 4 μm. Slides were deparaffinized in xylene and stained with hematoxylin/eosin (H&E; TissuePro Technology, Gainesville, FL, USA). Images were acquired using a slide scanner (Leica Microsystems, Wetzlar, Germany).

### 4.12. Statistical Analysis

All data are presented as mean ± standard deviation (SD) based on a minimum of three independent experiments. Statistical significance was assessed using one-way ANOVA, followed by Tukey’s post hoc test for multiple comparisons. A *p*-value of below 0.05 was considered to indicate statistical significance. All statistical analyses were conducted using GraphPad Prism version 9 (GraphPad software, San Diego, CA, USA).

## 5. Conclusions

This study provides evidence that TCE effectively ameliorates inflammation-induced peripheral edema through dual mechanisms, specifically enhancing endothelial barrier function and suppressing pro-inflammatory signaling pathways. The efficacy of TCE was demonstrated by its ability to decrease vascular permeability, inhibit leukocyte adhesion, and suppress systemic inflammatory responses through NF-κB and MAPK pathways. These findings highlight the potential of TCE as a promising candidate for the treatment of inflammation-associated vascular dysfunction and peripheral edema.

## Figures and Tables

**Table 1:** Representative compounds of TCE and their reported biological activities.

Compound	Chemical Structure	Reported Biological Activity	References
Chebulic acid	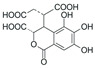	Prevents oxidative stress; protects endothelium from oxidative stress-induced dysfunction; supports vascular protection	[32,33]
Chebulinic acid	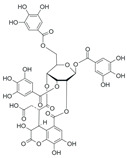	ROS scavenging; inhibits NF-кB phosphorylation; reduces pro-inflammatory mediators; blocks TNF-α-induced responses in infection	[34,35,36]
Chebulagic acid	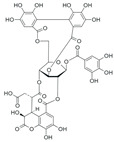	Strong antioxidant/anti-inflammatory; NF-кB and MAPK inhibition; reduces cytokine expression	[36,37]
Ellagic acid	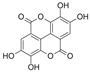	Inhibits NF-кB phosphorylation; reduces pro-inflammatory mediators; suppresses VEGF-induced migration in HUVECs	[38,39]
Gallic acid	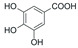	Canonical antioxidant; NF-кB and MAPK inhibition; reduces VEGF expression in endothelial cells	[36,40,41,42]
Corilagin	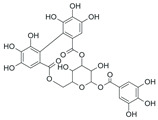	NF-кB and MAPK suppression; attenuates TNF-α-induced cytokine release and modulates VEGF expression	[43,44,45]

## Data Availability

The original contributions presented in this study are included in the article. Further inquiries can be directed to the corresponding authors.

## Abbreviations

The following abbreviations are used in this manuscript:

HUVECs	Human umbilical vein endothelial cells
*IL6*	Interleukin 6
MAPK	Mitogen-activated protein kinase
NF-κB	Nuclear factor kappa-light-chain-enhancer of activated B cells
*PTGS2*	Prostaglandin-endoperoxide synthase 2
ROS	Reactive oxygen species
TCE	*Terminalia chebula* extract
TEER	Transendothelial electrical resistance
*TNF*	Tumor necrosis factor
VEGF	Vascular endothelial growth factor
